# Mitochondrial Genome Characteristics Reveal Evolution of *Acanthopsetta nadeshnyi* (Jordan and Starks, 1904) and Phylogenetic Relationships

**DOI:** 10.3390/genes15070893

**Published:** 2024-07-08

**Authors:** Li-min Yang, Jing-feng Xue, Xiao-man Zhao, Ke Ding, Zhao-wen Liu, Zhou-si-yu Wang, Jian-bing Chen, You-kun Huang

**Affiliations:** 1 School of Materials and Environmental Engineering, Chizhou University, Chizhou 247000, China; yanglimin92@163.com (L.-m.Y.); liuzhaowen92@163.com (Z.-w.L.); 18815722702@163.com (Z.-s.-y.W.); chjb@czu.edu.cn (J.-b.C.); 2 Anhui Provincial Key Laboratory for Quality and Safety of Agri-Products, School of Resource and Environment, Anhui Agricultural University, Hefei 230036, China; jingfengxue@stu.ahau.edu.cn (J.-f.X.); zhaoxm@stu.ahau.edu.cn (X.-m.Z.); 3 State Key Laboratory of Mining Response and Disaster Prevention and Control in Deep Coal Mines, Anhui University of Science and Technology, Huainan 232001, China; dingke@cumt.edu.cn; 4 State Key Laboratory for Geomechanics and Deep Underground Engineering, China University of Mining and Technology, Xuzhou 221116, China

**Keywords:** mitogenome, divergence time, evolutionary pressure, phylogenetic construction

## Abstract

In the present study, the mitochondrial genomic characteristics of *Acanthopsetta nadeshnyi* have been reported and have depicted the phylogenetic relationship among Pleuronectidae. Combined with a comparative analysis of 13 PCGs, the TN93 model was used to review the neutral evolution and habitat evolution catalysis of the mitogenome to verify the distancing and purification selectivity of the mitogenome in Pleuronectidae. At the same time, a species differentiation and classification model based on mitogenome analysis data was established. This study is expected to provide a new perspective on the phylogenetic relationship and taxonomic status of *A. nadeshnyi* and lay a foundation for further exploration of environmental and biological evolutionary mechanisms.

## 1. Introduction

The data analysis of mitogenome is a fundamental indicator for analyzing the relationship between vertebrate evolution and phylogeny [1]. In recent years, these studies have provided a clearer positioning of species status on the ecological chain as more and more complete mitochondrial genome information is being reported [2,3]. Generally, the mitogenome structure of vertebrates is arranged in a specific order [4]. These components include 13 protein-coding genes (*ATP6*, *ATP8*, *COX1-3*, *CytB*, *NAD1-6*, and *NAD4L*), 2 ribosomal ribonucleic acids (*rrnS* and *rrnL*), and 22 transfer RNA genes (*tRNAs*) [5]. These characteristic information contain crucial information for molecular evolution, such as base composition bias, codon usage, and substitution rate [6]. In addition, the mitogenome with simple structures and low recombination levels exhibits more gene copies with fewer bases, making it widely used in the study of biological evolutionary origins and genetic diversity [7,8].

*A. nadeshnyi* (Jordan and Starks, 1904) (*Acanthopsetta*, Pleuronectiformes) is a species of ray-finned fish in the family of right-eye flounders. It is distributed in the northwest Pacific region, including the Korean Peninsula, Japan, and the Bering Sea [9,10]. In Pleuronectiformes, there are many species, but the genetic information of the reported species is limited. Several studies on comparative genomics are not sufficient to clarify the complete phylogenetic relationships [11]. Therefore, the ecological location of the species is not clear and there is an urgent need to carry out the ecological positioning of *A. nadeshnyi*.

With the development of molecular sequencing, comparative genomic results can no longer be a single indicator of species classification, and more reports will apply evolutionary data theory to the biological status of species [2,12,13]. In previous studies, different gene fragments have created different trees, but the complete evolutionary classification of this species is not clear: Studies have constructed phylogenetic trees for *COX1* and 16S rRNA of 10 species of Pleuronectiformes, but these two trees are not completely identical [14]; another study used the control region of the mitogenome as a sample to compare a different tree [15]. Ultimately, the incomplete mitogenome cannot exhibit consistent phylogenetic outcomes. A study reported the full length of the mitogenome sequences in *A. nadeshnyi*, but gene structure and evolutionary intervals have not been analyzed in conjunction with evolutionary trees unfortunately [16]. This situation leads to incomplete phylogenetic research and unclear ecological location of *A. nadeshnyi*. At the same time, the composition characteristics of the genome and the evolutionary trends of related species were analyzed, which will help to locate the ecological position of the species. In order to clarify the evolutionary relationships and states of species, we sequenced and assembled the entire mitochondrial gene of *A. nadeshnyi*. For the first time, the test of evolutionary divergence time provides a clearer elucidation of *A. nadeshnyis’* phylogenetic relationship and taxonomic status in this study.

## 2. Materials and Methods

### 2.1. Sample Collection, DNA Extraction, and PCR Amplification and Sequencing

The specimen of *A. nadeshnyi* was obtained from the east side of the Yellow Sea (33°47′ 10″ N, 126°59′ 30″ E) on 15 August 2017. This specimen (carcass length: 32 cm, female) is deposited in the Laboratory of the Museum of Materials and Environmental Engineering (Zhaowen Liu, liuzhaowen92@163.com), under voucher number AN2109073. The total genomic DNA was extracted using a modified phenol–chloroform method from the muscle tissue [3]. The sample was subjected to the Sanger sequencing method (Sangon Biotech, Shanghai, China), forming a circular mitochondrial genome. Design primers were based on published mitochondrial genomes and strictly amplified according to the requirements of the reagent kit (Takara, Beijing, China) (PrimeSTAR^®^ Max DNA Polymerase) (Supplementary Table S1).

### 2.2. Sequence Analysis and Assembly and Mitochondrial Genome Annotation

The sequencing fragment was spliced into a complete circular DNA using the CodonCode Aligner 5.1.5 (CodonCode Corporation, Dedham, MA, USA). We annotated the complete genome using a MITOS online server and manually corrected the tRNA structure (Supplementary Figure S1) [17].

### 2.3. Amino Acid Composition and Nucleotide Substitution Saturation Index of PCGs

The relative synonymous codon usage (RSCU) values and codon numbers were calculated by MEGA 11 [18]. The nonsynonymous mutation rates (Ka), synonymous mutation rates (Ks), and *Ka*/*Ks* ratio for the PCGs were calculated in DnaSP 5 [19]. The nucleotide substitution saturation index of PCGs was calculated using DAMBE, and the substitution fitting model for the three codons was TN93 [20].

### 2.4. Relative Evolutionary Rate Analysis

Mitochondrial genomes from 25 species were aligned using MUSCLE v3.8.31 (https://drive5.com/muscle/, accessed on 23 March 2024) and concatenated into a super-gene for each species. Evolutionary rates were determined from these super-gene sets using the tpcv module in the LINTRE program [21].

### 2.5. Divergence Time Estimation

The divergence times were estimated based on Beast 2 [22] via the approximate likelihood calculation method of the MCMCtree program, with the following parameters: --Substitution Model GTR; --Site Heterogeneity Model γ; --Tree Prior Yule Process; --Length of chain 10,000,000; and --Echo state to screen every 1000. Fossil records acquired from the TimeTree website (http://www.timetree.org) were used to calibrate the divergence times. We used TVBOT as a graphic beautification tool (https://www.chiplot.online/tvbot.html, accessed on 25 March 2024) [23].

### 2.6. Phylogenetic Tree Construction

Twenty-three complete Pleuronectidae mitochondrial genomes were downloaded from GenBank (https://www.ncbi.nlm.nih.gov/genbank/, accessed on 26 March 2024) for phylogenetic studies (Table 1). Two Saccopharyngiformes species, *Eurypharynx pelecanoides* and *Saccopharynx lavenbergi*, were selected as the outgroup. At the same time, in order to distinguish species and families, we set up the *Acipenser dabryanus* with a close relationship (Table 1). In the analysis, 13 PCG sequences were selected in order to construct a phylogenetic tree using MEGA 11 [18]. Ambiguous sequences were eliminated using Gblock v0.91 (Castresana, 2000) [24]. The phylogenetic analyses were conducted utilizing the MrBayes 3.2.6 and PhyML80 v3.0 software, based on Bayesian inference (BI) and maximum likelihood (ML), respectively [25,26]. ML analysis uses bootstrap analysis (1000 repetitions) to verify the relative support levels [27]. The resulting phylogenetic trees were visualized using FigTree v. 1.4.4 and its tool (https://itol.embl.de) [28].

## 3. Results and Discussion

### 3.1. Characteristics, Structure, and Overlapping of the Mitogenomes

**Figure 1:**
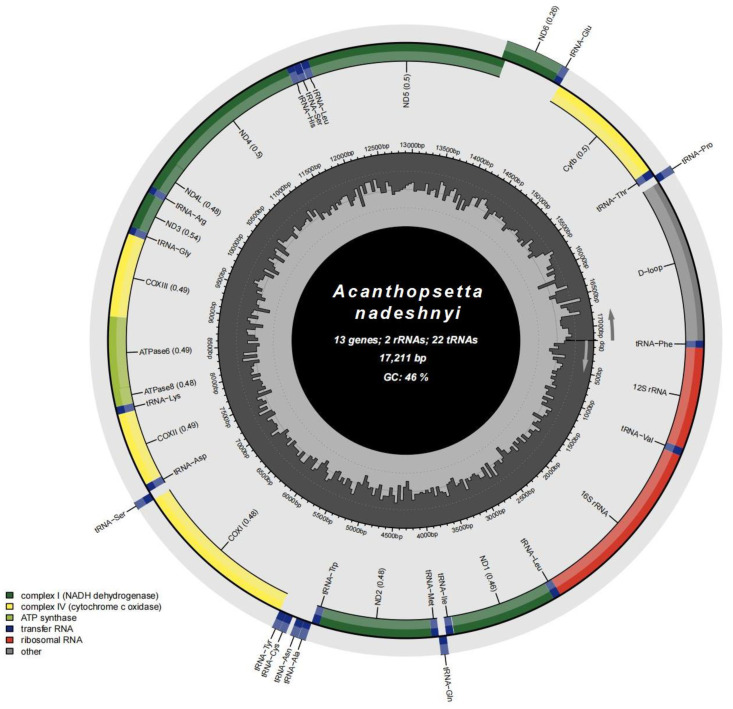
Gene map of *A. nadeshnyi* mitogenome.

Compared to the traditional mitogenomes, the mitochondrial genome of *A. nadeshnyi* exhibits the same gene sequence [29]. The complete mitogenome sequence of *A. nadeshnyi* was 17,211 bp (GenBank accessions OQ791285) (Figure 1). The circular mitochondrial genome contained 13 PCGs, 2 rRNA genes (12S rRNA and 16S rRNA), 22 putative tRNA genes, and a control region (Dloop).

In the mitogenomes, most of the coding fragments were on the heavy strand. Among them, eight tRNAs (*tRNA-Gln*, *tRNA-Ala*, *tRNA-Asn*, *tRNA-Cys*, *tRNA-Tyr*, *tRNA-Ser*, *tRNA-Glu*, and *tRNA-Pro*) were on the light strand, and the other fourteen tRNAs were all on the heavy strand. The length of each tRNA ranged from 65 to 74 bp (Table 2), and they were able to form a stable clover structure (Supplementary Figure S1). The small coding subunit (12S rRNA) and large coding subunit (16S rRNA) appeared on both sides with *tRNA-Phe* and *tRNA-Leu*, which were located on the H-chain and separated by the *tRNA-Val.* They were 950 bp and 1714 bp lengths, respectively (Table 2). Except for *ND6*, all remaining CD areas were located on the heavy strand (Figure 1).

### 3.2. Protein-Coding Genes and Codon Usage

The total length of all PCGs was 10596 bp in the mitogenome of *A. nadeshnyi,* which accounted for 61.57% of the whole genome (Table 2). The comparison of the initiation of all PCGs showed that all CDs start with ATG as the starting codon, except for *COX1* which was GTG. In terms of terminating codons, each CD was different: the *COX2*, *ND3*, *ND4*, and *Cytb* genes used an incomplete T stop codon; the *COX3* gene used TA; the *ND2* gene used TAG; and the *ND1*, *COX1*, *ATP8*, *ATP6*, *ND4L*, *ND5*, and *ND6* genes used TAA.

**Figure 2:**
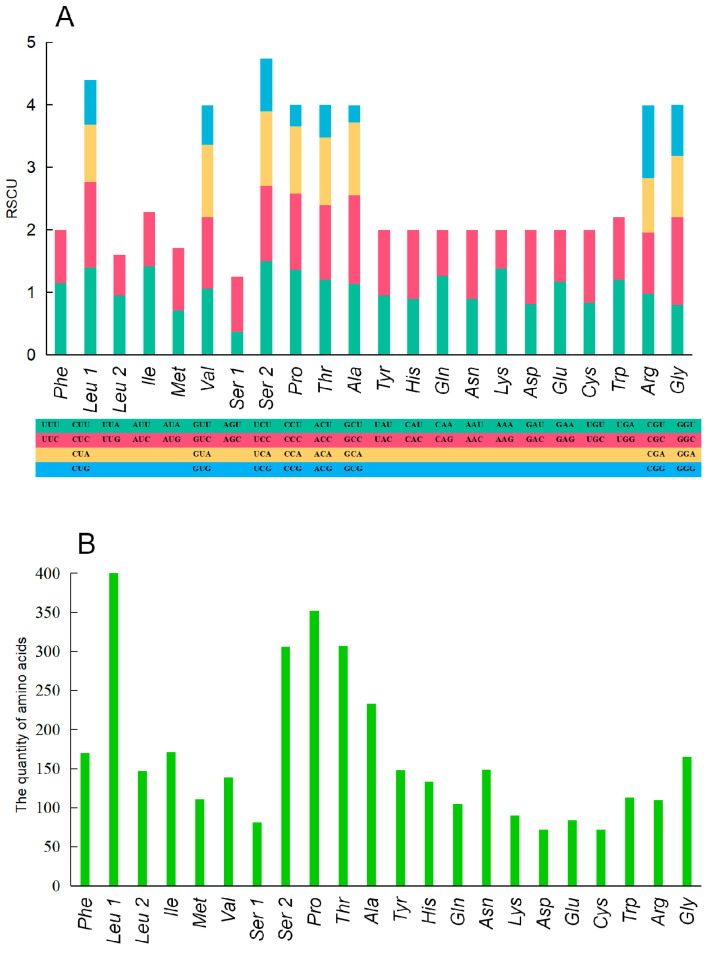
The relative synonymous codon usage (RSCU) in the mitogenome of *A. nadeshnyi* (**A**). (The y-axis represents the usage frequency of the corresponding amino acid codons in 13 PCGs. Different colors represent the different codons in the amino acids.) The amino acid composition in the mitogenome of *A. nadeshnyi* (**B**). (The x- and y-axis refer to the amino acid composition and the number of each amino acid in 13 PCGs, respectively.)

In an RSCU analysis, different codon usage frequencies indicated the selection evolutionary pressure of different amino acids (Figure 2). Generally speaking, the codon composition of longer amino acids is more abundant [30]. In this study, *Leu1*, *Ser 2*, *Pro*, and *Thr* also showed greater abundance compared to other amino acids. Interestingly, *Val*, *Arg*, and *Gly* exhibited a higher abundance with fewer quantities. Therefore, among multiple amino acid frequencies, *Val*, *Arg*, and *Gly* might also have a more stable genetic efficiency [31]. On the other hand, other amino acids may undergo relatively unstable genetic evolution due to genetic mutations or random genetic drift. These unstable sites may choose better amino acid codons due to different environmental selection pressures [30].

### 3.3. Mitogenome Mutations and Evolutionary Relationships in Pleuronectidae

**Figure 3:**
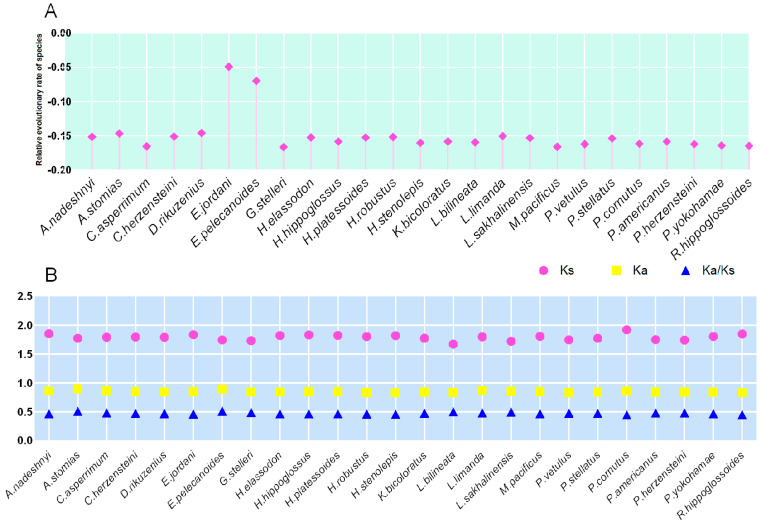
The relative evolutionary pressure index of species based on the mitochondrial genome model of Pleuronectidae (**A**). The ratio (Ks/Ks) of synonymous substitution (Ka) and synonymous substitution (Ks) calculated using amino acids as data points indicates the mutation pressure index of the mitochondrial genome (**B**).

In the publicly available mitogenomes, we selected twenty-three typical genomes of Pleuronectidae for calculating the evolutionary selection pressure of *A. nadeshnyi.* Meanwhile, we set up species *A. dabryanus* with the same close relationship and species *E. pelecanoides* and *S. lavenbergi* with two distant relationships to verify the accuracy of the results mutually. Based on the gene sequences of 13 PCGs of the mitogenomes, the relative evolutionary pressure of each species could be characterized. As shown in the results (Figure 3A), the evolutionary pressure on *E. jordani* and *E. pelecanoides* was relatively low. In other species, most stress indices were around −0.15, and this might be related to the genetic stability of mitochondria [32]. The relatively similar mutation pressure index not only demonstrates the maternal heritability of the mitogenomes but may also indicate the similarity of environmental selection pressure [33]. In the analysis of synonymous mutations in amino acids, different Ka and Ks values exhibit relatively similar *Ka*/*Ks* values (Figure 3B). The difference in Ka values among different Pleuronectidae species was relatively small, which may indicate that the frequency of neutral evolution was relatively similar among different species, and this accumulation of neutral evolution may also lead to non-environmental selectivity of the mitogenomes [34]. In the evolutionary process of the Pleuronectidae species, constrained evolution and divergent evolution seemed to be closely related to gene mutations (*Ka*/*Ks*). In most evolution, evolutionary selection of the mitogenomes eliminates harmful gene mutations and maintains the stability of the original amino acids [35].

**Figure 4:**
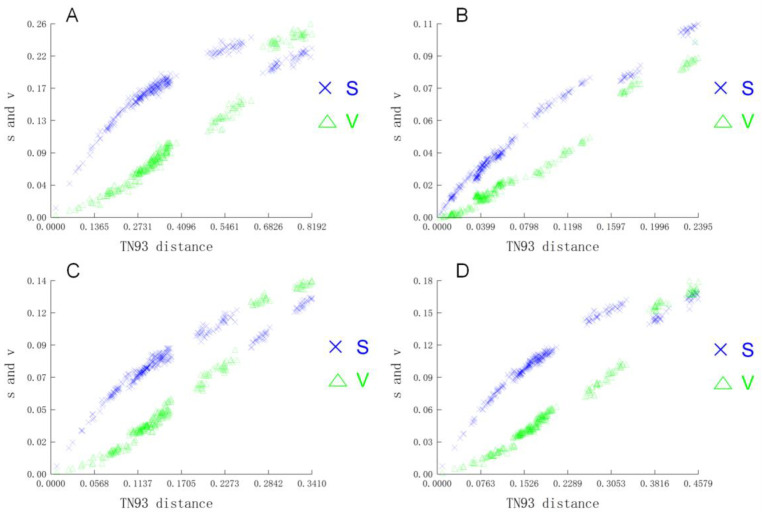
Nucleotide substitution saturation plots for all 13 PCGs of Pleuronectidae. First codon positions (**A**); second codon positions (**B**); third codon positions (**C**); and first codon and second codon positions (**D**). Plots in blue and green indicate transition and transversion, respectively.

**Figure 5:**
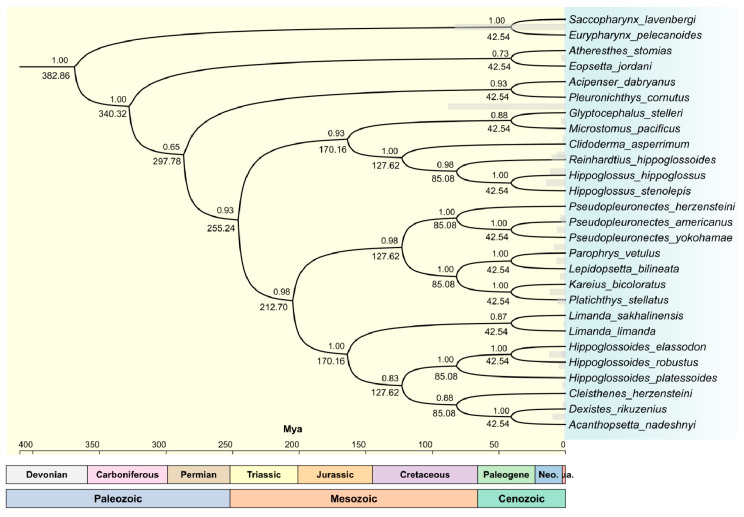
The divergence time and geological scale of species mitochondrial genomes of Pleuronectidae. The life evolution scale of this chart is based on the relative divergence time of two outer groups *E. pelecanoides* and *S. lavenbergi* as a unit. Among them, the divergence time between *E. pelecanoides* and *S. lavenbergi* is 25.9–118.3 Mya, and 39 Mya is selected as the unit scale based on species affinity (https://timetree.org).

At the same time, we used TN93 as a model to verify the base substitution ratio and nucleotide frequency of the three codons (Figure 4) [36]. Generally speaking, the nonsynonymy of the second codon (Figure 4B) and the synonymy of the third codon (Figure 4C) jointly affect changes in amino acid evolution [37]. In the results of this study, Figure 4B,C exhibited different enrichment patterns; the common frequency of the first and second codons (Figure 4D) was similar to the frequency characteristics of the first codon (Figure 5), and nonsynonymous mutations may approach neutral mutations. This result was mutually consistent with the above inference (*Ka*/*Ks*) and was also supported by the purify selection theory [38].

### 3.4. Divergence Time and Phylogenetic Analysis

In recent years, divergence time estimation has become increasingly prominent in evolutionary biology [39]. Methodological and empirical advances now allow time trees to be estimated more accurately than ever before [26]. It is assumed that the molecular evolution rate of a species is approximately constant, that is, the evolution rate of genetic differences should be proportional to the time of differentiation (molecular clock) [40]. For any large molecule (DNA sequence or protein sequence), there is an approximately constant evolutionary rate across all evolutionary lineages [41]. If the number of mutations aggregated on an evolutionary branch is proportional to the length of independent evolution time of that branch, then its replacement rate may approximately maintain a constant value during the evolution process [42]. Generally speaking, an accurate estimation method is to use the fossil time of a specific group as a correction, and then estimate the divergence time between species based on the degree of divergence between gene sequences and molecular clocks [43]. We can simultaneously estimate the occurrence time of other nodes on the phylogenetic tree in order to infer the origin of related groups and the divergence time of different groups [41,43].

In this study, all PCGs of the mitochondrial basic groups of twenty-seven species were used as units for calculating the evolutionary time tree (Figure 5). The life evolution scale of Figure 5 is based on the relative divergence time of two outer groups *E. pelecanoides* and *S. lavenbergi* as a unit. Among them, the divergence time between *E. pelecanoides* and *S. lavenbergi* is 25.9–118.3 Mya, and 39 Mya is selected as the optimal unit scale based on species affinity (https//timetree.org). It was interesting that species under the same subject cluster to other subjects. The clustering of most species of Pleuronectidae was natural, except for *A. stomias* and *E. jordani*. In the topology of divergence time (Figure 5), the separation time of *A. dabryanus* (Acipenseridae) was approximately 297.78–340.32 Mya, but it clustered together with species from other families. This may be convergent evolution of *A. dabryanus* and *P. cornutus* or other species of Pleuronectidae in similar habitats [44]. Perhaps due to the environment at different latitudes, light, and temperature, *A. stomias* and *E. jordani* underwent a divergent evolutionary process. Unfortunately, the comparison of mitochondrial genomes alone cannot be the sole explanation for this phenomenon, and it was necessary to correctly verify the survival range of all species worldwide.

Except for the divergence time of the cross-clustering, all species exhibited consistency in divergence time and sequence tree construction (Figure 5) (Supplementary Figure S2). The tree construction of the two results showed almost similar clustering topology. *A. nadeshnyi* and *D. rikuzenius* differentiated over a period of 42.54 Mya during the Cenozoic (Figure 5), and similar conclusions were also reflected in the results of systematic development (Supplementary Figure S2). The differentiation time of Pleuronectidae was 42.54–340.32 Mya, and this may be related to the changes in Quaternary glacier movement [45,46,47]. Unfortunately, due to the ancient nature of the mitogenome, the results of gene tree construction may be different from those of the species tree.

## 4. Conclusions

In this study, we reported the complete mitochondrial genome of *A. nadeshnyi*, analyzed the corresponding genomic information, compared with the reported mitogenomes of *A. nadeshnyi* and congeneric species, and depicted the phylogenetic relationship among Pleuronectidae. Meanwhile, combined with a comparative analysis of 13 PCGs, the TN93 model was used to review the neutral evolution and environmental evolution catalysis of the mitochondrial genome to verify the distancing and purification selectivity of the mitochondrial genome in Pleuronectidae. A cross-analysis model for species differentiation and tracing was established using mitochondrial genome data using divergence time and phylogenetic analysis. In future research, more mitochondrial genome data will be made public, and this data model will be more accurate when combined with the historical changes in the coastline. This study provides basic data support for analyzing the genetic data features of *A. nadeshnyi*, while also providing a more theoretical basis for the evolutionary classification of Pleuronectidae.

## Figures and Tables

**Table 1:** Species attribution and accession number.

Species	Species	Family	Order	Accession No.
*A. nadeshnyi*	*A. nadeshnyi*	Acanthopsetta	Pleuronectidae	OQ791285
*Atheresthes stomias*	*A. stomias*	Atheresthes		NC_083173
*Cleisthenes herzensteini*	*C. herzensteini*	Cleisthenes		NC_028021
*Clidoderma asperrimum*	*C. asperrimum*	Clidoderma		MK210570
*D. rikuzenius*	*D. rikuzenius*	Dexistes		NC_066467
*Eopsetta jordani*	*E. jordani*	Eopsetta		NC_083049
*Glyptocephalus stelleri*	*G. stelleri*	Glyptocephalus		NC_060723
*Hippoglossoides elassodon*	*H. elassodon*	Hippoglossoides		NC_082804
*Hippoglossoides platessoides*	*H. platessoides*			MN122825
*Hippoglossoides robustus*	*H. robustus*			NC_082769
*Hippoglossus hippoglossus*	*H. hippoglossus*	Hippoglossus		NC_009709
*Hippoglossus stenolepis*	*H. stenolepis*			NC_009710
*Kareius bicoloratus*	*K. bicoloratus*	Kareius		NC_080271
*Lepidopsetta bilineata*	*L. bilineata*	Lepidopsetta		NC_083649
*Limanda limanda*	*L. limanda*	Limanda		OY755015
*Limanda sakhalinensis*	*L. sakhalinensis*			NC_082768
*Microstomus pacificus*	*M. pacificus*	Microstomus		NC_082805
*Parophrys vetulus*	*P. vetulus*	Parophrys		OR482580
*Platichthys stellatus*	*P. stellatus*	Platichthys		NC_010966
*Pleuronichthys cornutus*	*P. cornutus*	Pleuronichthys		NC_022445
*Pseudopleuronectes americanus*	*P. americanus*	Pseudopleuronectes		NC_082555
*Pseudopleuronectes herzensteini*	*P. herzensteini*			NC_063673
*Pseudopleuronectes yokohamae*	*P. yokohamae*			NC_028014
*Reinhardtius hippoglossoides*	*R. hippoglossoides*	Reinhardtius		NC_009711
*A. dabryanus*	*A. dabryanus*	Acipenser	Acipenseridae	NC_036420
*E. pelecanoides*	*E. pelecanoides*	Eurypharynx	Eurypharyngidae	AB046473
*S. lavenbergi*	*S. lavenbergi*	Saccopharynx	Saccopharyngidae	AB047825

**Table 2:** Features of mitochondrial genomes of *A. nadeshnyi*.

Mitogenome	Position		Length	Amino	Start/Stop	Intergenic Region from to (bp) *	Strand ^#^
	From/To		(bp)	Acid	Codon		
*tRNA-Phe (F)*	1	68	68			0	H
*12S RNA*	68	1017	950			−1	H
*tRNA-Val (V)*	1018	1090	73			0	H
*16S RNA*	1091	2804	1714			0	H
*tRNA-Leu^UUA^ (L1)*	2807	2880	74			2	H
*ND1*	2881	3855	975	325	ATG/TAA	0	H
*tRNA-Ile (I)*	3861	3930	70			5	H
*tRNA-Gln (Q)*	4000	3930	69			−1	L
*tRNA-Met (M)*	4001	4069	69			0	H
*ND2*	4069	5115	1047	349	ATG/TAG	−1	H
*tRNA-Trp (W)*	5114	5185	72			−2	H
*tRNA-Ala (A)*	5255	5187	69			1	L
*tRNA-Asn (N)*	5329	5257	73			1	L
*tRNA-Cys (C)*	5431	5367	65			37	L
*tRNA-Tyr (Y)*	5500	5433	68			1	L
*COX1*	5502	7061	1560	520	GTG/TAA	1	H
*tRNA-Ser^UCA^ (S1)*	7132	7062	71			0	L
*tRNA-Asp (D)*	7147	7217	71			14	H
*COX2*	7224	7914	691	230	ATG/T	6	H
*tRNA-Lys (K)*	7915	7987	73			0	H
*ATP8*	7989	8156	168	56	ATG/TAA	1	H
*ATP6*	8147	8830	684	228	ATG/TAA	-10	H
*COX3*	8830	9614	785	261	ATG/TA	-1	H
*tRNA-Gly (G)*	9615	9686	72			0	H
*ND3*	9687	10035	349	116	ATG/T	0	H
*tRNA-Arg (R)*	10036	10104	69			0	H
*ND4L*	10105	10401	297	99	ATG/TAA	0	H
*ND4*	10395	11775	1381	460	ATG/T	−7	H
*tRNA-His (H)*	11776	11845	70			0	H
*tRNA-Ser^AGC^ (S2)*	11846	11912	67			0	H
*tRNA-Leu^CUA^ (L2)*	11919	11989	71			6	H
*ND5*	11990	13840	1851	617	ATG/TAA	0	H
*ND6*	14321	13803	519	173	ATG/TAA	−38	L
*tRNA-Glu (E)*	14390	14322	69			0	L
*Cyt b*	14395	15535	1141	380	ATG/T	4	H
*tRNA-Thr (T)*	15536	15608	73			0	H
*tRNA-Pro (P)*	15678	15608	71			−1	L
Dloop	15679	17211	1533			0	H

* Intergenic region: non-coding bases between the feature on the same line and the line below, with a negative number indicating an overlap. ^#^ H: heavy strand; L: light strand.

## Data Availability

The data presented in this study are available to researchers eligible under the Research Ethics Board rules on request from the corresponding author due to ethical restrictions.

## Supplementary Materials

The following supporting information can be downloaded at: https://www.mdpi.com/article/10.3390/genes15070893/s1, Figure S1: The cloverleaf structure of 22 tRNAs in the mitochondrial genome of A.nadeshnyi; Figure S2: Phylogenetic tree of the Pleuronectidae inferred from the nucleotide sequences of 13 PCGs using the Bayesian inference (BI) and maximum likelihood (ML) methods. The numbers on the branches indicate: (1) Evolutionary Branch Length Values: phylogenetic relationships after species establishment, (2) Bootstrap: the confidence value of kinship distance; Table S1: PCR amplification steps; Table S2: PCR amplification primers.
