# Supplementary figures and images for: Mitochondrial Genome Characteristics Reveal Evolution of Acanthopsetta nadeshnyi (Jordan and Starks, 1904) and Phylogenetic Relationships
Source: Genes (Basel). 2024 Jul 8;15(7):893. doi: 10.3390/genes15070893 (PMC11276143; doi:10.3390/genes15070893)

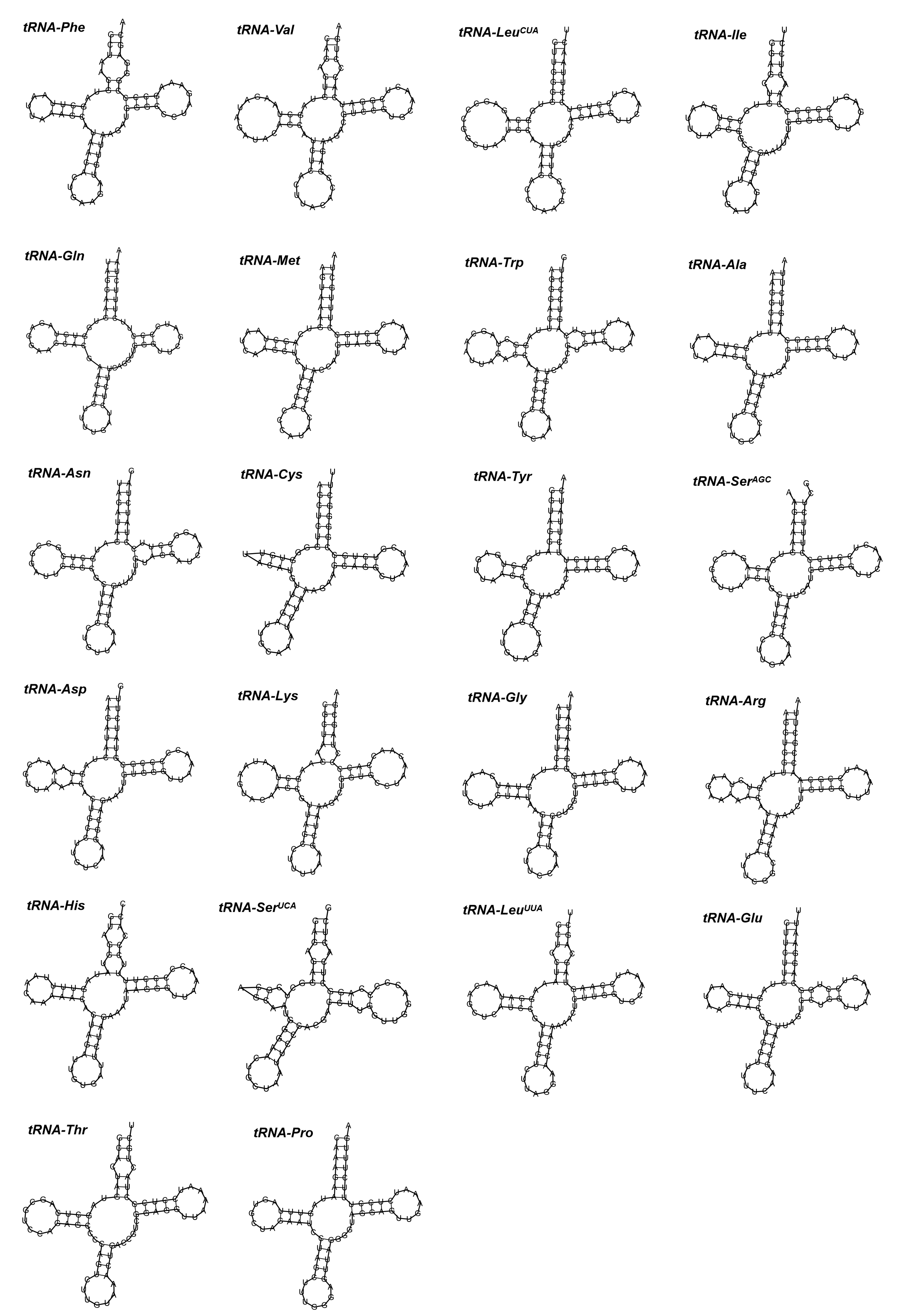

Supplement: Supplementary file 1 [file genes-15-00893-s001.zip › Supplementary/Supplementary Fig.1.png]

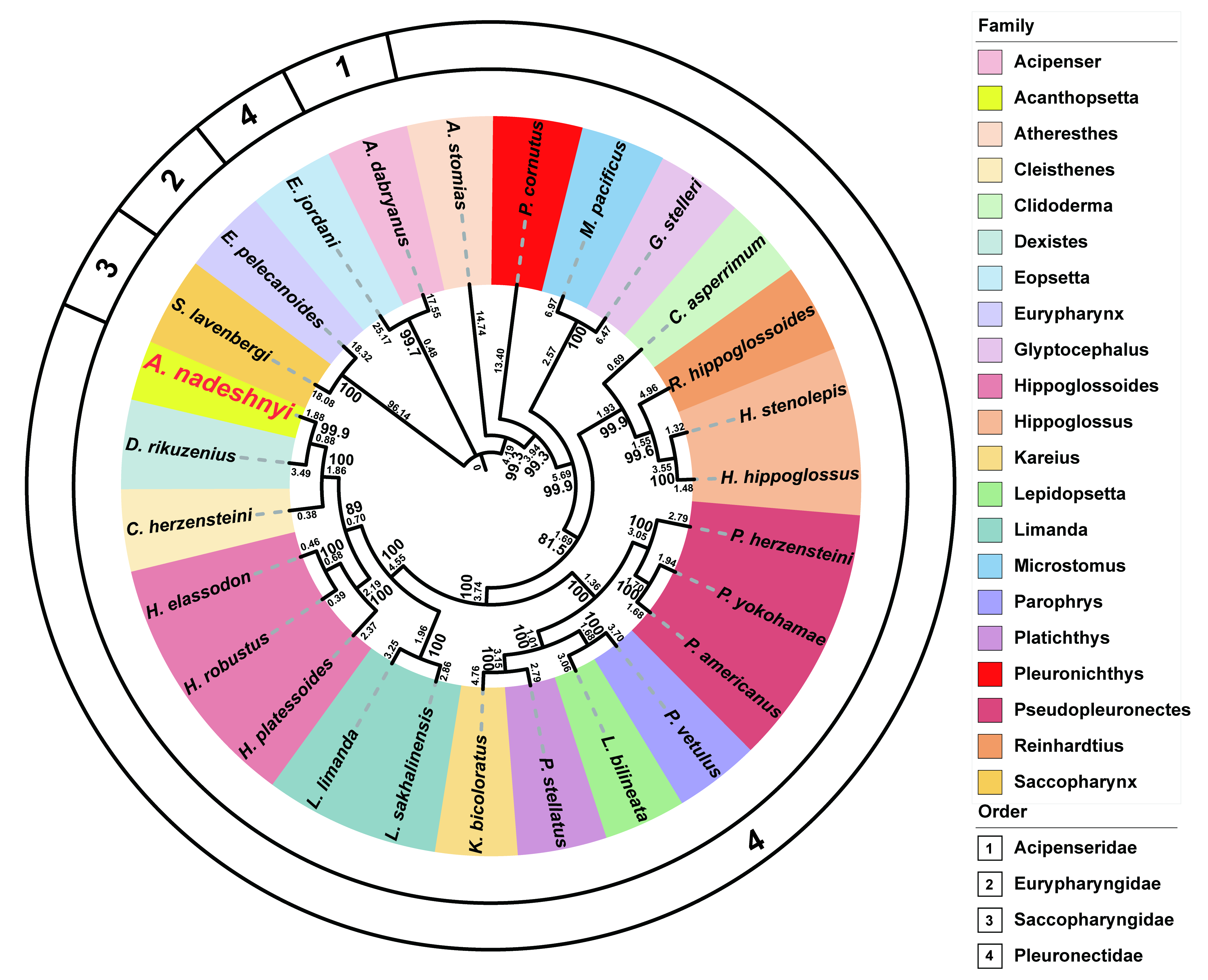

Supplement: Supplementary file 1 [file genes-15-00893-s001.zip › Supplementary/Supplementary Fig.2.tif]
